# Identification of miR-199a-5p, miR-214-3p and miR-99b-5p as Fibrosis-Specific Extracellular Biomarkers and Promoters of HSC Activation

**DOI:** 10.3390/ijms22189799

**Published:** 2021-09-10

**Authors:** Catherine Jane Messner, Saskia Schmidt, Dilek Özkul, Carine Gaiser, Luigi Terracciano, Stephan Krähenbühl, Laura Suter-Dick

**Affiliations:** 1 School of Life Sciences, University of Applied Sciences and Arts Northwestern Switzerland, Hofackerstrasse 30, CH-4132 Muttenz, Switzerland; saskia.schmidt@fhnw.ch (S.S.); dilek.oezkul@gmail.com (D.Ö.); carine.gaiser@fhnw.ch (C.G.); 2 Department of Pharmaceutical Sciences, University of Basel, CH-4001 Basel, Switzerland; Stephan.Kraehenbuehl@usb.ch; 3 Swiss Centre for Applied Human Toxicology (SCAHT), CH-4001 Basel, Switzerland; 4 Institute of Pathology, University Hospital Basel, CH-4001 Basel, Switzerland; Luigi.Terracciano@usb.ch

**Keywords:** liver fibrosis, microRNA, biomarkers, 3D in vitro model, drug-induced liver injury

## Abstract

Liver fibrosis is characterized by the accumulation of extracellular matrix (ECM) resulting in the formation of fibrous scars. In the clinic, liver biopsies are the standard diagnostic method despite the potential for clinical complications. miRNAs are single-stranded, non-coding RNAs that can be detected in tissues, body fluids and cultured cells. The regulation of many miRNAs has been linked to tissue damage, including liver fibrosis in patients, resulting in aberrant miRNA expression/release. Experimental evidence also suggests that miRNAs are regulated in a similar manner in vitro and could thus serve as translational in vitro–in vivo biomarkers. In this work, we set out to identify and characterize biomarkers for liver fibrosis that could be used in vitro and clinically for research and diagnostic purposes. We focused on miRNAs released from hepatic 3D cultures exposed to methotrexate (MTX), which causes fibrosis, and acetaminophen (APAP), an acute hepatotoxicant with no clinically relevant association to liver fibrosis. Using a 3D in vitro model, we corroborated compound-specific responses as we show MTX induced a fibrotic response, and APAP did not. Performing miRNA-seq of cell culture supernatants, we identified potential miRNA biomarkers (miR-199a-5p, miR-214-3p, niRNA-125a-5p and miR-99b-5p) that were associated with a fibrotic phenotype and not with hepatocellular damage alone. Moreover, transfection of HSC with miR-199a-5p led to decreased expression of caveolin-1 and increased α-SMA expression, suggesting its role in HSC activation. In conclusion, we propose that extracellular miR-214-3p, miR-99b-5p, miR-125a-5p and specifically miR-199a-5p could contribute towards a panel of miRNAs for identifying liver fibrosis and that miR-199a-5p, miR-214-3p and miR-99b-5p are promoters of HSC activation.

## 1. Introduction

Liver fibrosis is characterized by the accumulation of extracellular matrix (ECM), which distorts hepatic architecture through the formation of fibrous scars. The relevant cellular and molecular events leading to scar formation are described in the adverse outcome pathway (AOP) and include hepatocellular death/injury, macrophage activation, TGF-β1 expression, stellate cell activation and deposition of ECM [1]. If left untreated, liver fibrosis ultimately progresses into cirrhosis, resulting in hepatocellular dysfunction and reduced intrahepatic blood flow, leading to hepatic insufficiency and portal hypertension [2,3]. Liver cirrhosis can also promote the development of hepatocellular carcinoma (HCC) [4]. Accurately diagnosing early stages of liver fibrosis is essential for disease management, due to the reversibility of fibrosis through the removal/treatment of the cause of injury [5,6,7]. A leading cause of liver damage involves the intake of drugs, known as drug-induced liver injury (DILI), which is a major challenge in clinical medicine and drug development [8]. Certain drugs such as methotrexate (MTX) elicit chronic DILI in some patients and can lead to hepatic fibrosis and ultimately cirrhosis [9,10]. Other drugs, such as acetaminophen (APAP), are safe at recommended pharmacological doses but cause acute, dose-related DILI when overdosed. APAP hepatotoxicity is characterized by hepatocellular necrosis/apoptosis and liver failure that can be resolved or be fatal, but there are no reported clinical cases of APAP-induced liver fibrosis [8,11].

Currently the most trusted method of diagnosing liver fibrosis is a liver biopsy, which is still considered the standard diagnostic method despite being invasive and carrying the risk of clinical complications [12]. Non-invasive methods for identifying liver damage are well-established and include serum biomarkers such as alanine aminotransferase (ALT), aspartate aminotransferase (AST), alkaline phosphatase (ALP), albumin levels and total bilirubin (TBL) [13]. These biomarkers are indicative of hepatocellular damage and cholestatic liver injury, but are not specific to the diagnosis of fibrosis. Combinations of serum markers and other parameters (e.g., demographic and blood cell count) produce scores, which can be used for diagnosis of liver injury, examples of these tests include APRI, fibrosis-4 or Fibroindex [13]. Additional diagnostic alternatives include ultrasonography, computed tomography, magnetic resonance and transient elastography [14,15,16,17]. These methods detect a variety of liver diseases, lack sensitivity, can be expensive and involve radiological risks [13,14,18]. Thus, the quest for early and specific biomarkers of liver fibrosis is still ongoing.

Small regulatory RNAs known as microRNAs (miRNAs) are single-stranded, non-coding RNAs consisting of 21–25 nucleotides. miRNAs are not only located intracellularly but can be released and circulate in a variety of bio-fluids (e.g., plasma, serum and urine) and cell culture media [19]. It has been shown that a variety of diseases such as cancer result in selective miRNA release, making miRNAs promising potential biomarkers for specific diseases [20,21,22,23,24,25]. Extracellular miRNAs are released via extracellular vesicles, protein complexes or lipoproteins and mediate cell–cell signaling [19,26]. An example of intercellular communication from secreted miRNAs was shown by Zhang et al. who demonstrated miR-150 was selectively packed into microvesicles, which were actively secreted and able to enter and deliver miR-150 to an immortalized human microvascular endothelial cell line, leading to a downregulation of c-Myb expression and increased cell migration [27]. There have also been studies to identify potential miRNAs that modulate cell phenotype during liver fibrosis, examples include miR-146b, miR-221 and miR-222. These miRNAs are linked to HSC activation in human liver or cell culture samples [28,29,30,31]. However, Krauskopf et al. demonstrated that miR-222 release is associated with patients experiencing APAP overdose, showing that miR-222 would be unsuitable as a non-invasive fibrosis marker [32]. Some miRNAs (e.g., miR-33a, miR-181b and miR-17) have been linked to HSC activation and their release is increased extracellularly in patients with fibrosis elicited by chronic hepatitis B or non-alcoholic fatty liver disease [33,34,35,36,37]. Yet, none of these miRNAs have been studied in the context of DILI-induced fibrosis and been confirmed to be fibrosis-specific.

Several miRNAs have been identified as potential DILI biomarkers in clinical samples, in vivo and in vitro models [25,32,38,39,40,41,42,43], including liver-specific miR-122-5p that is predominantly expressed by hepatocytes and released into the blood upon hepatocellular injury. APAP-induced liver injury is an example of DILI-elicited liver injury and miR-122-5p release [25,41,42,43,44]. A recent publication reported increased release of miR-122 from a bio-printed multicellular human model exposed to MTX [45]. Moreover, miR-122-5p was released in exosomes by 3D-HepaRG cells treated with MTX, before the onset of toxicity, demonstrating the sensitivity of miRNAs as early biomarkers [44]. Despite miR-122 being liver-specific and promising for identifying liver injury, its release from cells has been mainly associated with hepatocellular damage and is not specific to liver fibrosis.

Many miRNAs associated with the progression of liver fibrosis are typically measured intracellularly. Some examples include miR-199, miR-200, miR-2861, miR-33a and many more [46]. However, measuring intracellular miRNAs requires tissue collection and therefore entails an invasive procedure (e.g., liver biopsy). Circulating miRNAs would be more suitable biomarkers for in vivo and in vitro studies, as they can be detected non-invasively. A small number of circulating miRNAs such as miR-34a, miR-19b, miR-33a, miR-27a, miR-17, miR-125a and miR-29 have shown aberrant release during fibrosis progression caused by NAFLD, HBV and HCV [47,48].

Scaffold-free multicellular 3D liver microtissues (MTs), comprising HepaRG, THP-1 and hTERT-HSC (surrogates for hepatocytes, Kupffer cells and hepatic stellate cells, respectively), have been shown to recapitulate key fibrotic events elicited by known pro-fibrotic stimuli such as TNF-α, TGF-β1 and pro-fibrotic compounds MTX and thioacetamide (TAA) [49]. In the present study, we aimed at identifying miRNAs specifically released by MTs with a fibrotic phenotype. To this end, we exposed MTs to APAP or MTX, in order to differentiate hepatocellular damage from fibrosis. We used next-generation sequencing (NGS) to identify miRNAs released into the cell culture supernatant and corroborated putative specific biomarkers with qPCR. Furthermore, we wanted to elucidate whether the identified miRNAs would also play a regulatory role in the physiology of the stellate cell. Using miRNA mimics in combination with hTERT-HSCs, we assessed the effects of the selected miRNAs on HSC activation.

## 2. Results

### 2.1. The 3D In Vitro Liver Model Maintains Metabolic Activity and Can Develop a Fibrotic Phenotype

**Figure 1:**
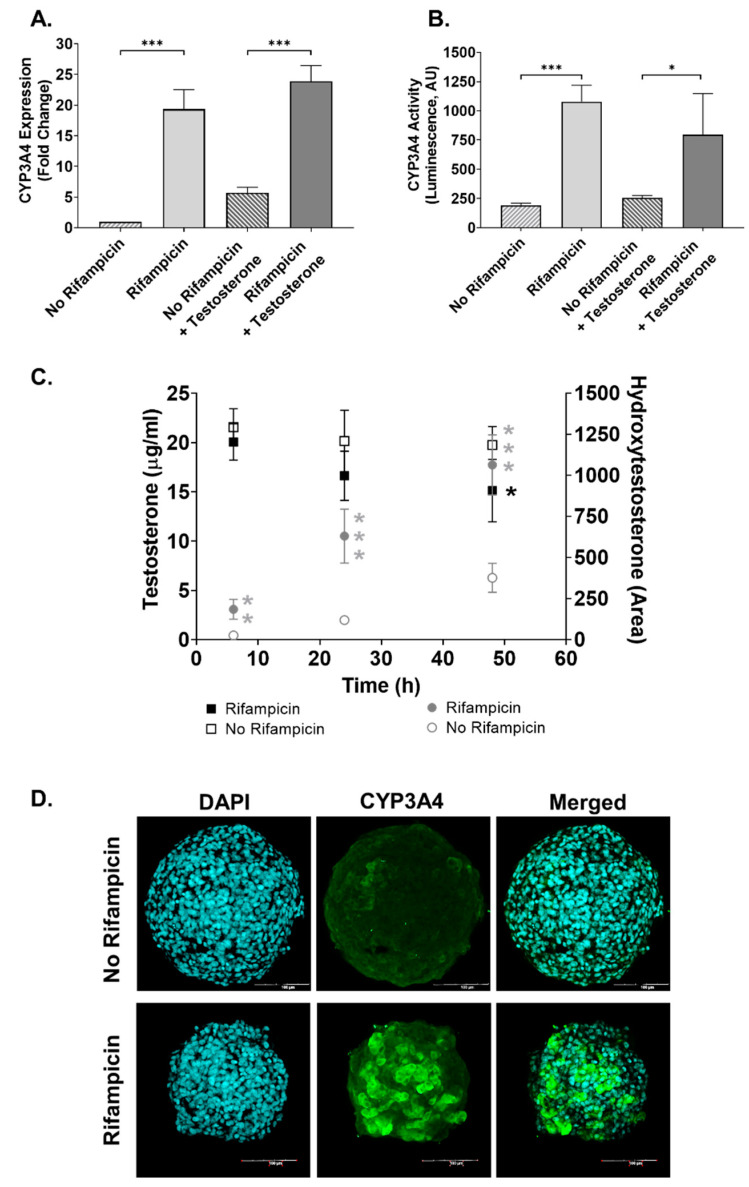
Characterization of CYP3A4 activity in 3D human liver MTs. MTs were exposed to 20 µM rifampicin (RIF), and RIF-treated and untreated MTs were exposed to 25 µg/mL testosterone for a further 48 h. Expression of CYP3A4 was determined by q-RT-PCR, expressed as fold change in comparison with the control; *n* = 3 (**A**). CYP3A4 activity was measured by a P450-GloTM CYP3A4 assay and expressed in luminescence arbitrary units (AU); *n* = 9 (**B**). Conversion of testosterone (25 µg/mL) into hydroxytestosterone (OH-testosterone) at 6, 24 and 48 h was determined by HPLC-MS/MS. Data are expressed as concentrations (µg/mL) calculated using a testosterone calibration curve. Area under the curve in arbitrary units (AU) was used for calculating OH-testosterone; *n* = 6 (**C**). Representative image of immunohistochemical detection of CYP3A4, scale bar = 100 µm (**D**). Bar graphs represent means ± SD; statistical analysis based on Student’s unpaired *t* test; boxplot represents means ± SD; statistical analysis based on Student’s unpaired *t* test for rifampicin vs. no rifampicin for each timepoint; *, *p* ≤ 0.05; **, *p* ≤ 0.01; ***, *p* ≤ 0.001.

The ability to bio-transform xenobiotics is key for the liver and should be displayed by HepaRG cells in 3D cultures. To evaluate this in our system, we measured CYP3A4 expression and activity in MTs 48 after the aggregation period without (basal) and with induction with rifampicin (RIF). Expression of CYP3A4 was detected under basal conditions and was strongly induced in MTs treated with RIF (Figure 1A). Enzymatic activity of CYP3A4 was also detectable under basal conditions and significantly induced by rifampicin. This is supported by results obtained with the P450-GloTM CYP3A4 assay (Figure 1B) and by measuring the metabolic transformation of testosterone to hydroxytestosterone using LC-MS (Figure 1C). Immunostaining of the MTs (Figure 1D) shows the marked induction elicited by rifampicin and the uniform distribution across the MT of CYP3A4 protein expression, further supporting basal and inducible expression of CYP3A4. Moreover, we confirmed CYP2E1 expression in our MTs for up to two weeks in culture (Figure S1).

**Figure 2:**
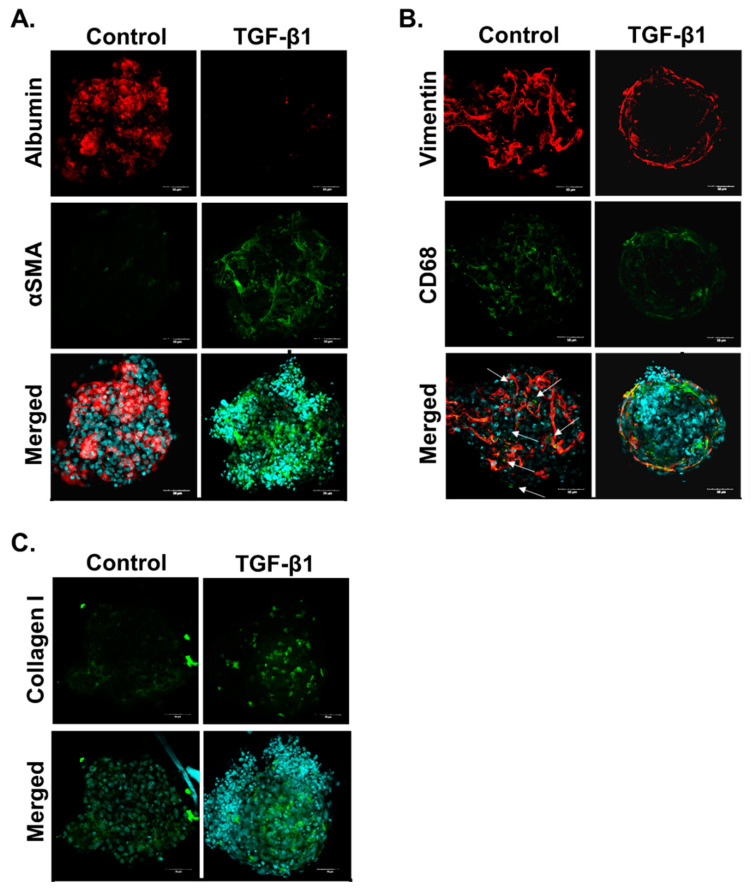
TGF-β1 induces fibrosis in human liver MTs. MTs were left untreated or exposed to 1 ng/mL TGF-β1 for 10 days which was refreshed every 2–3 days. The MTs were fixed and stained to demonstrate localization of the three cell types: HepaRG cells stain positive for albumin; hTERT-HSC stain positive for αSMA and vimentin; and THP-1 stain positive for CD68 and vimentin. HSC activation and ECM deposition were shown through increased αSMA and collagen I staining, respectively. Images are shown as a maximum intensity projection including the merged image with DAPI for each staining combination: albumin and αSMA (**A**), vimentin and CD68 (**B**) and collagen (**C**). Arrows identify THP-1 as these cells are positive for both CD68 and vimentin (**B**).

In addition to HepaRG cells remaining metabolically active, immunostaining of untreated MTs confirmed that the three cell types are present in the MTs 10 days after aggregation (13 days in total from seeding). All three cell types are required to develop a relevant fibrotic response. Immunostaining of MTs showed expression of specific protein markers: HepaRG express albumin (Figure 2A); hTERT-HSC express the intermediate filament vimentin (Figure 2B); and THP-1 express vimentin and CD68 (Figure 2B). The cells in the MTs also displayed a fibrotic response as shown by their reaction to treatment with the pro-fibrotic cytokine TGF-β1 (1 ng/mL) for 10 days. TGF-β1 treatment elicited a reduction of albumin stain, indicating hepatocellular damage probably to HepaRG cells (Figure 2A). It also led to increased protein expression of αSMA and collagen I (Col I), showing that HSCs have become activated and ECM production has increased, thereby demonstrating TGF-β1 can promote a fibrotic phenotype in the MTs (Figure 2B,C).

### 2.2. Methotrexate but Not Acetaminophen Induces Fibrosis in the MTs

**Figure 3:**
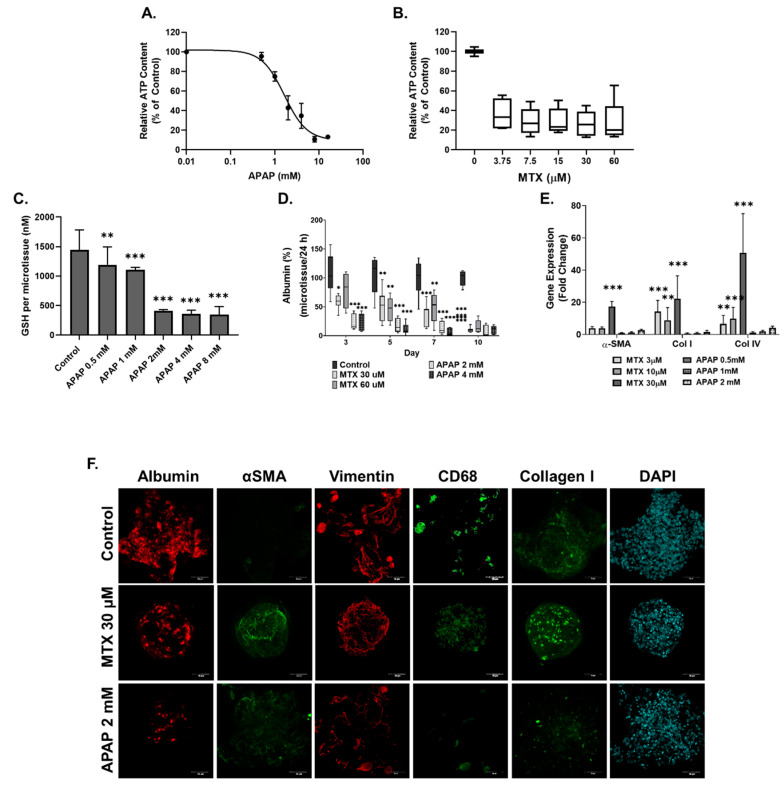
MTX and APAP elicit compound-specific responses in human liver MTs. MTs were exposed to MTX and APAP for 10 days, which were refreshed every 2-3 days. Viability was assessed for APAP- (**A**) and MTX (**B**)-treated MTs using the CellTiterGlo luminescence kit and expressed as relative ATP content (% of control), *n* = 9. IC50 for APAP was calculated to be 1.7 ± 0.38 mM but IC50 for MTX was not able to be calculated accurately. GSH was measured using a GSH-Glo™ Assay for APAP-treated MTs, *n* = 9. (**C**). Albumin release was measured for treated MTs using albumin ELISA and shown as percentage, *n* = 9 (**D**). Gene expression of αSMA, Col I and Col IV was measured using q-RT-PCR in treated MT samples; untreated microtissues were used as control (fold change 1), *n* = 6 (**E**). MTs were fixed and stained for albumin, αSMA, vimentin and CD68. HSC activation and ECM deposition were shown through increased αSMA and collagen I staining, respectively. Images are shown as a maximum intensity projection for each staining (**F**). Bar graphs and boxplots represent means ± SD; statistical analysis based on one-way ANOVA (**C**) and Student’s unpaired *t* test (**D**,**E**); *, *p* ≤ 0.05; **, *p* ≤ 0.01; ***, *p* ≤ 0.001.

MTs were treated with MTX (3.75–60 µM) and APAP (0.5–16 mM) for 10 days. At the end of the treatment period, cellular ATP content was determined as a measure of cell viability. The EC50 was calculated for APAP and found to be 1.7 ± 0.38 mM, whereas MTX resulted in a 60–70% decrease in ATP content for all concentrations (Figure 3A,B). Cellular glutathione (GSH) was also measured in APAP-treated MTs as detoxification of APAP is expected to lead to GSH depletion. In our model, APAP exposure at 2 mM and above led to a large decrease in GSH (Figure 3D). Based on the EC50 of APAP and the GSH results, we determined 2 and 4 mM to be appropriate for investigating APAP-induced hepatotoxicity. MTX concentrations 30 and 60 µM were selected for further investigation based on previous experiments described by Prestigiacomo et al. showing that these concentrations could elicit fibrosis in human liver MTs [50]. Damage by the compounds to the HepaRG cells was assessed by measuring albumin production. Both concentrations of APAP and MTX resulted in decreased albumin release but the onset of the effect differed: while APAP reached its maximum effect at day three, MTX showed a more gradual effect, indicating an acute and chronic response, respectively (Figure 3D).

Evidence of fibrosis in the MTs after ten days exposure to both toxicants was investigated using q-RT-PCR and immunostaining. q-RT-PCR results show that MTX induced a significant increase in Col I and Col IV expression at all concentrations. Significant αSMA expression was only seen in MTs treated with 30 µM MTX. APAP showed no significant increase in αSMA, Col I and Col IV expression for the toxic 2 mM concentrations (i.e., overdose) and sub-toxic 0.5 mM concentration of APAP. Finally, immunostaining results show albumin decrease for the MTX and APAP treatments, which confirm both treatments cause damage to the albumin-producing HepaRG cells. However, both compounds differ in their effect on fibrosis markers: while MTX increased αSMA and Col I expression, APAP did not.

### 2.3. Identification of Fibrosis-Specific miRNAs

**Figure 4:**
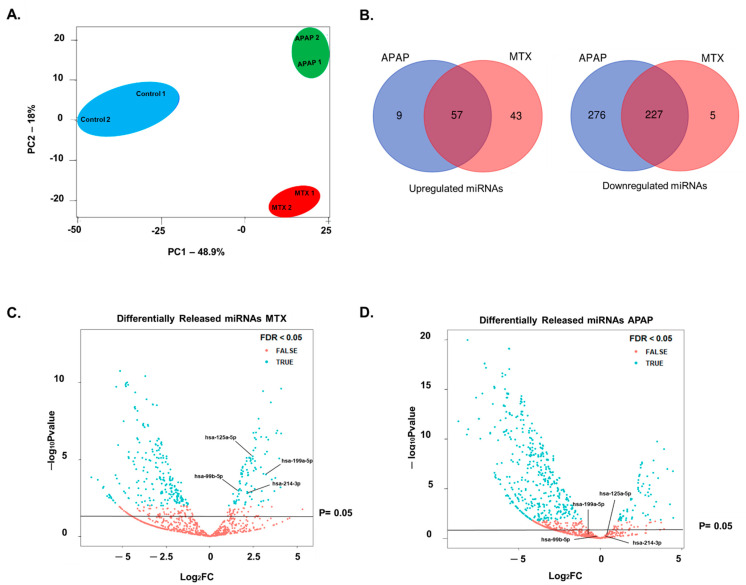
Using NGS to investigate miRNAs released by MTs treated with MTX and APAP. Cell culture supernatant from MTs exposed to MTX and APAP for 10 days were collected to perform miRNA analysis using NGS. PCA analysis of differentially released miRNAs was carried out using R as described in methods section (**A**). NGS demonstrated that MTX and APAP differentially released miRNAs. Venn diagram results show that release was increased for 57 and decreased for 225 common miRNAs between the treatments (**B**). Four of the MTX-specific miRNAs were chosen based on the literature search and volcano plots for MTX (**C**) and APAP (**D**) demonstrate the release of the four miRNAs (miR-199a-5p, mIR-214-3p, miR-125a-5p and miR-99b-5p) is significantly increased by MTX treatment but not APAP.

Based on the results described above, we performed further investigation of the differing responses of MTs treated with a pro-fibrotic (MTX) and a hepatotoxicant that was not a pro-fibrotic (APAP). In order to identify fibrosis-specific markers, we collected cell culture supernatant from the final 72 h of the 10-day exposure period, when fibrosis was evident in the MTX-treated MTs. All small RNAs, including miRNAs, piRNAs and tRNAs, in the medium were analyzed by NGS. Preliminary analysis of NGS data using principal component analysis (PCA) demonstrated that the control, MTX and APAP samples grouped separately. More specifically, the change in small RNA release by treated (MTX and APAP) MTs were discriminated from untreated samples in principal component 1 (PC1). This is likely due to the common toxic effects of both compounds. The more interesting differences between MTX- and APAP-treated samples are represented in the second principal component (PC2), where the effects of MTX and APAP differ (Figure 4A).

By assessing the differentially released small RNAs for each treatment we identified nine small RNAs that were released specifically due to APAP exposure and 43 small RNAs that were released specifically due to MTX treatment. The latter may be associated with liver fibrosis (Figure 4B).

Using these 43 small RNAs, we removed piRNA and tRNAs and focused only on the miRNAs. Further refinement of the selection of putative miRNAs markers was based on known links to fibrosis or wound healing from in vitro, in vivo and/or clinical studies, on an extensive literature search (summarized in Table 1) and by using DIANA miRpath v3.0. This process led to the choice of four miRNAs for further investigation (miR-199a-5p, miR-214-3p, miR-125a-5p and miR-99b-5p). The differential release of these miRNAs by MTX- and APAP-treated MTs is depicted in Figure 4C,D.

**Figure 5:**
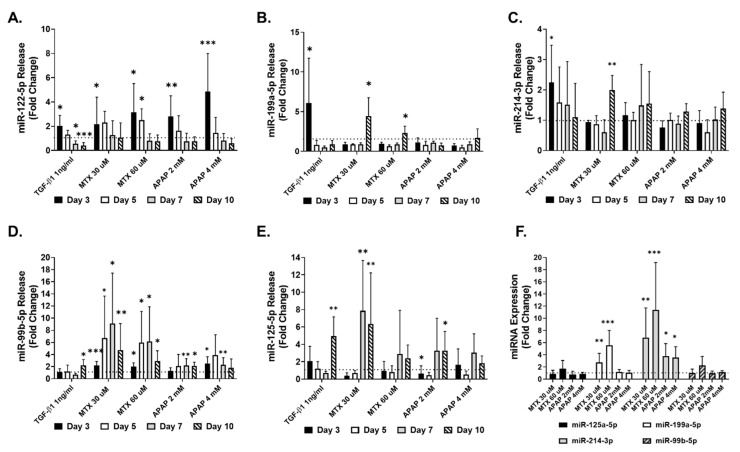
miRNA corroboration using q-RT-PCR. MTs were exposed to TGF-β1, MTX or APAP for 10 days and cell culture supernatant was collected to investigate miRNA release using q-RT-PCR. Release of miR-122-5p (**A**), miR-199a-5p (**B**), miR-214-3p (**C**), miR-99b-5p (**D**) and miR-125a-5p (**E**) was measured at day 3, day 5, day 7 and day 10, and analyzed using q-RT-PCR. miRNA was also measured intracellularly to assess changes in miR-125a-5p, miR-199a-5p, miR-214-3p and miR-99b-5p expression at day 10 (**F**). Data are expressed as fold change compared to untreated control (fold change 1); *n* = 9 for A, C, D and E. Bar graphs represent means ± SD; statistical analysis based on Student’s unpaired *t* test; *, *p* ≤ 0.05; **, *p* ≤ 0.01; ***, *p* ≤ 0.001.

Further investigations of the four selected miRNA species and miRNA-122-5p, a known marker of hepatocellular damage, included confirmatory independent experiments to demonstrate their consistent release into the medium determined by q-RT-PCR. The results of these measurements are depicted in Figure 5A–E). The pro-fibrotic positive control TGF-β1 elicited an increase in miR-122a-5p, miR-199a-5p and miR-214-3p at the earliest measured time point (Figure 5A–C). In contrast, miR-99b-5p and miR-125a-5p increased at later time points (Figure 5D,E). All the miRNAs, with the exception of miR-199a-5p and miR-122-5p, were released to a lesser extent by the TGF-β1 treatment in comparison to the MTX and/or APAP (Figure 5A–E). As expected, miR-122a-5p was released upon exposure to both MTX and APAP due to the compounds’ toxicity to the MTs. APAP caused a more transient increase (at day three), while MTX lead to an increase in miRNA-122-5p in the medium at days three and five, consistent with their respective acute and chronic toxicity mechanisms (Figure 5A). The predominant release of the selected four miRNAs by MTs treated with MTX was corroborated in these independent experiments and the evaluation over several time points uncovered additional kinetic differences. For each of the four miRNAs tested, the release from MTX-treated samples was higher than that from APAP-treated samples. For example, miR-99b-5p release was increased upon exposure to MTX 30 and 60 µM at all time points, reaching an approximately eight-fold higher release, while APAP led to a maximum increase of approximately three-fold. Similar effects were observed for the other miRNAs. This is also evident for miR-199-5p (at day 10), miR-214-4p (MTX 30 µM, at day 10), miR-99-5p (at days five and seven) and miR-125-5p (MTX 30 µM, at days seven and ten). Taken together, these results confirm the findings obtained in the previous experiment using NGS on the differential release of the four selected miRNAs. It also shows that the effects of MTX generally appear later, with minimal changes at the early time point (day three). Finally, the intracellular miRNA levels were investigated using q-RT-PCR, demonstrating that only the expression of miR-199a-5p and miR-214-3p was significantly increased upon exposure to 30 and 60 µM MTX, while miR-125a-5p and miR-99b-5p remained unchanged. APAP elicited a significant increase in miR-214-3p expression but did not affect the expression of miR-125a-5p, miR-199a-5p and miR-99b-5p (Figure 5F).

### 2.4. Translation from Human Cell Lines to Human Primary Cells

**Figure 6:**
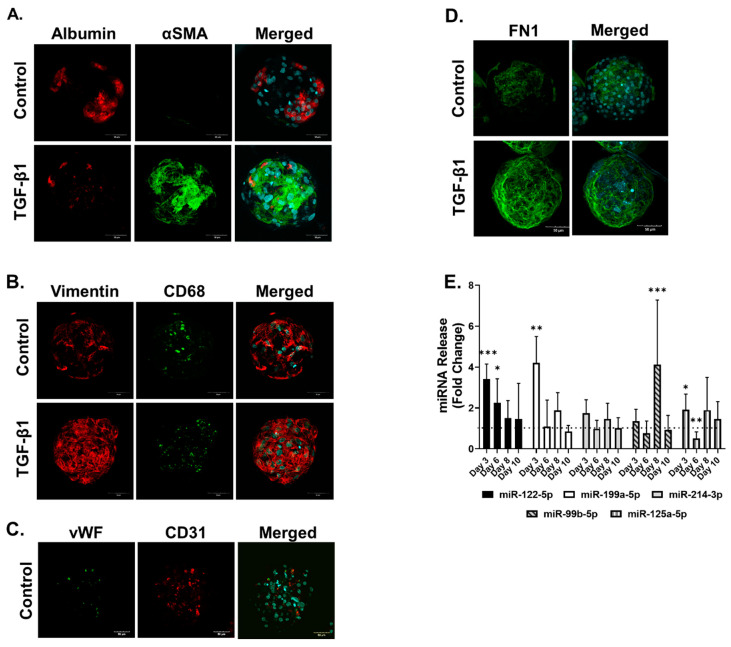
Measurement of miRNAs in primary MTs exposed to TGF-β1. Primary MTs were left untreated or exposed to TGF-β1 for 3-10 days. Primary MTs were fixed at day 10 and co-stained for αSMA and albumin (**A**), vimentin and CD68 (**B**), CD31 and vWF (**C**) and FN1 (**D**), and counterstained with DAPI. Cell culture supernatant was collected over 10 days to investigate miRNA release of primary MTs exposed to TGF-β1 using q-RT-PCR. miRNAs measured included: miR-122-5p, miR-199a-5p, miR-214-3p, miR-99b-5p and miR-125a-5p, which were measured at day 3, day 6, day 8 and day 10, and analyzed using q-RT-PCR (**E**). Data are expressed as fold change, *n* = 9. Bar graphs represent means ± SD; statistical analysis based on Student’s unpaired *t* test; *, *p* ≤ 0.05; **, *p* ≤ 0.01; ***, *p* ≤ 0.001.

**Figure 7:**
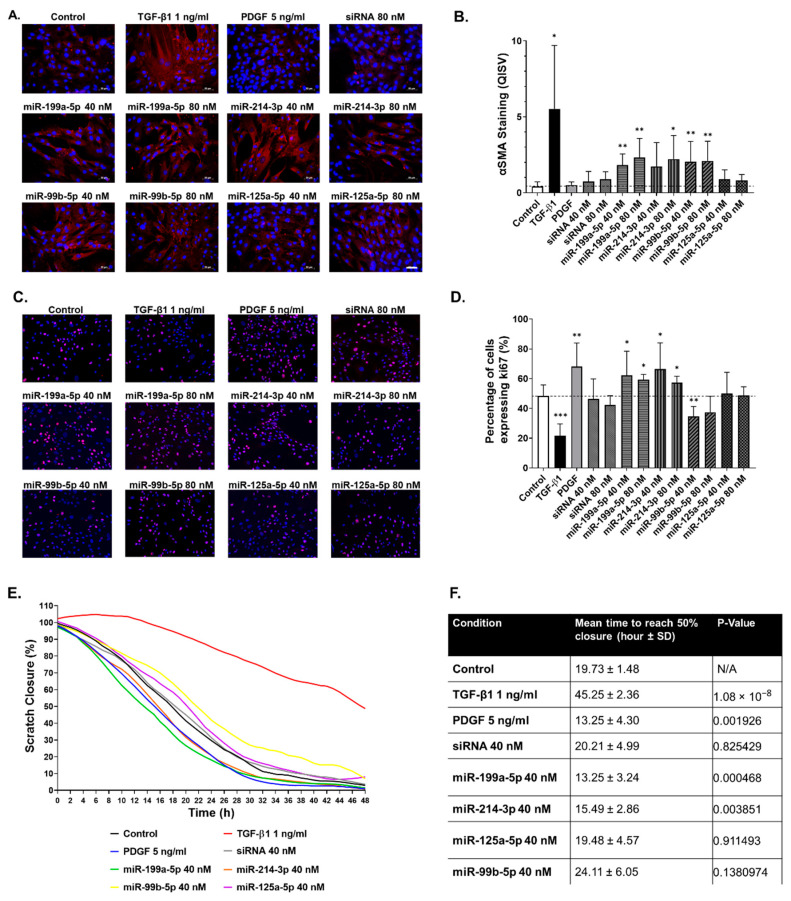
miR-199a-5p, miR-214-3p and miR-99b-5p promote hTERT-HSC activation. hTERT-HSC were transfected with the four mimics and an siRNA negative control (40 and 80 nM) for 72 h and were either fixed or used for migration assessment. αSMA staining was carried out to investigate the potential of the 4 miRNA mimics to elicit HSC activation, which were compared to positive controls TGF-β1 and PDGF (**A**) and quantified using ImageJ (**B**). Proliferation was assessed using ki67 staining (**C**), and was also quantified using ImageJ (**D**). Migration was assessed using a migration assay that was imaged every hour over 48 h and quantified using ImageJ to assess the speed of wound closure (**E**). The time for each condition to reach 50% wound closure was assessed to calculate significance between migration capacities for each condition (**F**). *n* = 6 for immunostaining quantification. *n* = 8–9 for the migration assay. Student’s unpaired *t* test; *, *p* ≤ 0.05; **, *p* ≤ 0.01; ***, *p* ≤ 0.001.

Primary human microtissues (PHMTs) were generated using a similar process to that for the cell line model (see Materials and Methods). Characterization of cellular composition based on albumin, vimentin and CD68 immunostaining confirmed the successful incorporation of hepatocytes, HSCs and Kupffer cells (KCs) into the PHMT. PHHs stained positive for albumin (Figure 6A), both HSCs and KCs stained positive for vimentin (Figure 7B), and endothelial cells stained positive for CD31 and von Willebrand (vWF) (Figure 6C). KCs were distinguished from HSCs by CD68 staining within the PHMTs (Figure 6B). TGF-β1 (1 ng/mL) treatment of PHMTs elicited hepatocellular damage and HSC activation demonstrated by the reduction in albumin staining and increased αSMA-staining, respectively (Figure 6A). We also confirmed ECM deposition through the increased FN1 staining in the treated PHMTs (Figure 6D).

Cell culture supernatant was collected over 10 days to identify whether the four miRNAs of interest and miRNA-122-5p were increased by TGF-β1-induced fibrosis in the PHMTs (Figure 6E). q-RT-PCR results show that miR-122-5p was significantly increased at days 3–6 by the TGF-β1 treatment, demonstrating the expected hepatocellular damage. We also identified a significant increased release of three of the four putative fibrosis markers, miR-199a-5p, miR-125a-5p and miR-99b-5p. miR-214-5p release increased to a lesser extent, corresponding to the cell-line-based model where miR-214-3p showed the smallest increase of the four miRNAs (Figure 6E). These results demonstrate the relevance and transferability of the findings from the cell-line-based model to the primary model.

### 2.5. Functional Impact of Selected miRNAs on hTERT-HSC

Following identification of the four miRNAs in the MT model and confirmation that they are preferentially released upon exposure to MTX, we set out to investigate their functional effect on HSC to expand on the literature search results described in Table 1. To this end, we transfected miRNA mimics into hTERT-HSC grown in monolayers and assessed their effect on functional responses related to HSC activation. The effects of the miRNA mimics on HSC activation status were assessed by immunostaining for αSMA in the transfected cells (Figure 7A). The pro-fibrotic cytokine TGF-β1 caused a large increase in αSMA protein and the formation of stress fibers and was used as positive control throughout these experiments (Figure 7A,B). The growth factor PDGF is also involved in wound healing and plays a role in promoting migration/proliferation, but does not promote increased αSMA in HSCs (Figure 7A,B). Three of the four miRNAs (miR-199a-5p, miR-214-3p and miR-99b-5p) led to a significant increase in αSMA protein, while miR-125a-5p did not (Figure 7A,B). We also evaluated the functional response of the hTERT-HSC in terms of cell migration and proliferation, key features of HSC during fibrosis. Consistent with previous data, TGF-β1 treatment resulted in a decrease in proliferation shown by ki67 staining, whereas PDGF resulted in a significant increase in proliferation (Figure 7C,D). Transfection with miR-199a-5p and miR-214-5p also resulted in a significant increase in proliferation, whereas miR-99b-5p reduced the quantity of ki67 staining. miR-125a-5p and siRNA had no effect on hTERT-HSC proliferation (Figure 7B,C).

The effect of transfection on the migration capacity of the hTERT-HSC was assessed hourly for 48 h, starting 72 h after transfection. Similar to the proliferation results, TGF-β1 treatment decreased and PDGF increased the migration capacity of hTERT-HSC (Figure 7E,F). Results also show that that migration was accelerated by miR-199a-5p and miR-214-3p transfection (Figure 7E,F). miR-99b-5p, miR-125a-5p and negative control siRNA did not significantly affect hTERT-HSC migration (Figure 7E,F).

### 2.6. miR-199a-5p Targets Caveolin-1 in hTERT-HSCs

**Figure 8:**
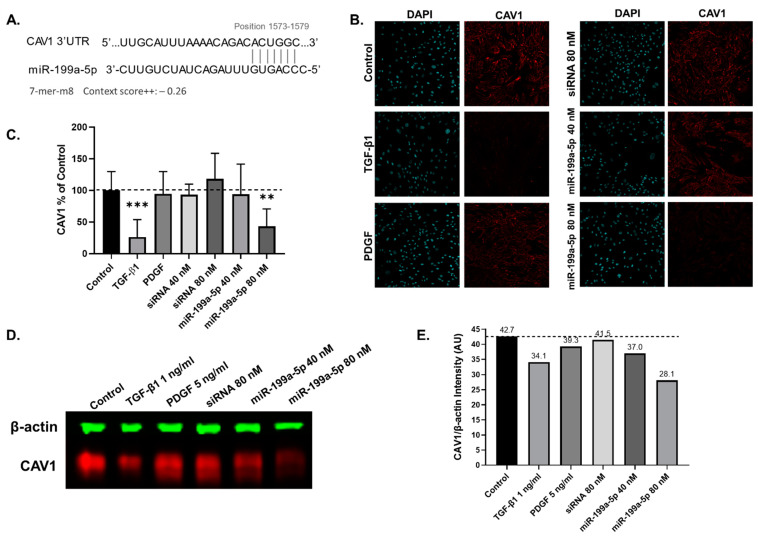
Identification of potential targets of the four selected miRNAs. Following the literature review, CAV1 was identified as a potential target for miR-199a-5p and was confirmed using TargetScan (accessed on 7 June 2021), where miR-199a-5p was found to target CAV1 on positions 1573–1579 with an exact match to positions 2–8 of the mature miRNA. The context score++ is −0.26, of which 1 = lowest repression capacity and −1 = highest repression capacity (**A**). hTERT-HSC were transfected for 72 h with miR-199a-5p mimic and were fixed or collected in RIPA buffer at 72 h. Potential target CAV1 was assessed using immunostaining, showing a decrease in TGF-β1-treated and miR-199a-5p-transfected hTERT-HSCs, which was confirmed following quantification using ImageJ (**B**,**C**). These findings were corroborated using Western blot analysis (**D**), which were quantified using the Li-Cor Image Studio to obtain intensity (AU) of CAV1/β-actin (**E**). Student’s unpaired *t* test; **, *p* ≤ 0.01; ***, *p* ≤ 0.001.

miR-199a-5p appears to be a promising fibrosis-specific marker based on the miRNA release and expression experiment. Upon exposure to pro-fibrotic stimuli, but not to APAP, miR-199a-5p displayed the largest increase in extracellular release in both the cell line and primary MTs (Figure 5B and Figure 6E) and the strongest intracellular induction in the cell line MTs (Figure 5F). We also show that miR-199a-5p is involved in HSC activation as it increases αSMA production and promotes migration and proliferation (Figure 7). For this reason, we investigated the potential downstream effects of miR-199a-5p in the hTERT-HSCs to better understand the link between miR-199a-5p and fibrosis progression. Using DIANA-miRpath v3. and subsequently TargetScan, we identified caveolin-1 (CAV1) as a potential target. TargetScan results show miR-199a-5p has an exact match to positions 2–8 of the mature miRNA (7-mer-m8) to position 1573–1579 on CAV1 with a context score^++^ of −0.26 (Figure 8A). Following the 72 h transfection, the hTERT-HSCs were either fixed for immunostaining or lysed in RIPA buffer for Western blot analysis. Once again, TGF-β1 and PDGF were used as positive controls for HSC activation. Immunostaining results show TGF-β1 elicited a visible decrease in CAV1 staining (Figure 8B,C). miR-199a-5p-80 nM-transfected hTERT-HSCs had significantly decreased CAV1 staining, whereas miR-199a-5p 40 nM decreased it to a lesser extent (Figure 8B,C). These results were confirmed using Western blot analysis, which shows decreased CAV1 protein in the TGF-β1-treated sample and the miR-199a-5p-transfected samples in comparison to the control, PDGF and siRNA that show no big decrease in CAV1 quantity (Figure 8D,E).

## 3. Discussion

### 3.1. Characterisation of Human Liver MTs

The capacity of hepatocytes to metabolize xenobiotics is a key hepatic function mostly catalyzed by CYP450s. CYP3A4 is the most abundant isoform in the human liver, responsible for the metabolism of a large number of clinically used drugs (>50%) [60]. RIF is capable of transcriptionally inducing CYP3A4 and is a common method used to assess CYP induction and metabolic capacity of hepatocytes in vitro [44,61,62,63]. In accordance with our previous studies using a 3D HepaRG model, this cell line displays basal and inducible CYP3A4 activity in the MT [44]. In addition, CYP2E1, the main cytochrome responsible for APAP metabolism, is expressed in our model. This is confirmed by its expression level and by the observed APAP-induced toxicity. These results agree with published data by us [64] and others [65] showing that HepaRG in 3D culture display CYP2E1 expression and metabolic capacity, respectively. Furthermore, we confirmed that the MTs display other typical hepatic characteristics such as albumin production [66,67]. THP-1 and hTERT-HSC were identified by CD68 and vimentin staining, confirming successful incorporation of all three cells lines, as previously reported by Prestigiacomo et al. [49] TGF-β1 is a key cytokine in liver fibrosis progression and is described as a key event (KE) in the AOP for liver fibrosis. These key events include hepatocellular death/injury (KE1), KC activation and macrophage recruitment (KE2), TGF-β1 expression (KE3), HSC activation (KE4) and collagen accumulation (KE5) [1]. Thus, the pro-fibrotic effect of TGF-β1 is downstream from the hepatocellular injury as it directly activates the HSC via the Smad-signaling cascade [68]. The ability of the 3D human liver MTs to develop a fibrotic phenotype in response to TGF-β1 was confirmed by the increased αSMA and FN1 expression. Taken together, these results show that this MT model represents a suitable in vitro alternative for the study of metabolism and liver fibrosis progression.

### 3.2. Compound-Specific Response of MTs Exposed to MTX and APAP

High concentrations of APAP lead to acute hepatocellular necrosis/apoptosis and liver failure (overdose) [8,11], whereas MTX elicits chronic DILI in some patients, potentially leading to hepatic fibrosis and ultimately cirrhosis [9,10]. Considering that fibrosis develops over time in a chronic-injury setting in the clinic and in in vitro systems [2,9], we focused our investigation on the adverse outcomes in the MTs exposed to both these compounds after long-term exposure (10 days).

As described in our previous study, suitable concentrations of the test compounds MTX and APAP were determined based on the known C_max_ (maximal drug concentration in human plasma), where it has been justified that in vitro studies may use a dose up to 100 × C_max_ and is described in more detail in Messner et al., 2020 [44]. Therefore, we conclude that 30 and 60 µM MTX are appropriate for in vitro fibrosis studies, despite being higher than the C_max_ in patients. APAP elicited significant, acute and hepatocellular responses with an EC50 of 1.7 ± 0.38 mM, similar to that shown by others using PHHs (EC50 range 1.5–2.5 mM) after 7 days of exposure [69]. Hepatocellular damage was substantiated by reduced albumin release and increased release of miR-122-5p measured at day three. The effects of APAP in the MT also mimicked the GSH depletion caused in patients by N-acetyl-p-benzoquinoneimine (NAPQI) accumulation following APAP overdose [11,70]. MTX, on the other hand, caused hepatotoxicity in the MTs in a more gradual fashion, mimicking chronic damage, displaying decreased albumin release and increased miR-122-5p release, and ultimately leading to a fibrotic phenotype (increased αSMA and Col I). These findings are in agreement with in vitro data investigating hepatotoxicity and/or fibrosis [45,49,71,72] and with clinical findings from psoriasis patients [9,73]. In summary, this in vitro model is sensitive to MTX- and APAP-induced hepatotoxicity, and can differentiate between MTX-induced fibrosis and APAP-induced acute toxicity, as seen clinically.

### 3.3. Extracellular miRNAs as Potential Markers of Fibrosis

Based on the response of the MTs to APAP and MTX, we hypothesized that the two compounds could be used to identify biomarkers able to differentiate acute hepatocellular necrosis/apoptosis from DILI-induced fibrosis. We focused on differentially released miRNAs identified using NGS and a subsequent database (DIANA miRPath v3.0) and literature analysis, excluding in particular miRNAs known to be released in patients who have suffered an APAP overdose [32]. The chosen panel consisted of miR-199a-5p, miR-214-3p, miR-99b-5p and miR-125a-5p. miR-199a has been shown to be increased in liver fibrosis biopsy samples and the severity of liver fibrosis correlated with increased expression of miR-199a [29,46,52]. Additionally, TGF-β1 has been shown to upregulate miR-199a-3p in HSCs [74]. Our data suggest that miR-199a-5p is a suitable candidate as a fibrosis-specific miRNA, as we show that intracellular expression and extracellular release of miR-199a-5p was induced by MTX, but not by APAP. Intracellular miR-214 has also been reported to be increased during liver fibrosis and HSC activation [46,53,75]. We not only identified increased miR-214-3p expression; we also show an increase in extracellular levels upon exposure to MTX.

A correlation has been identified between miR-125a and HBV-induced liver fibrosis, HCV-associated hepatotoxicity and HSC activation [54,55,56]. We detected a large and significant increase in miR-125a-5p release upon exposure to MTX 30 µM and only a small increase by APAP 2 mM. Finally, miR-99b has been linked to pericellular fibrosis from NAFLD and wound healing/regeneration due to dermal and spinal cord injury [57,58,59]. Extracellular levels of miR-99b-5p increased upon exposure to both MTX and APAP. Interestingly, MTX led to sustained miR-99b-5p release (i.e., chronic release), as both concentrations elicited a significant increase in release at all the times measured. In comparison, the increase caused by APAP was much smaller and less consistent over time.

To rule out the possibility that the detected effect was specific to the used cell lines, we exposed PHMTs to the pro-fibrotic factor TGF-β1 and assessed their responses. We confirmed the fibrotic phenotype was induced by TGF-β1 by hepatocellular injury (decrease in albumin staining and increased miR-122-5p release) and by HSC activation and ECM deposition (increased αSMA and FN1 staining) in primary cells, FN1 being a crucial ECM component produced alongside Col I during liver fibrosis progression [2]. Under the tested conditions, TGF-β1-treated PHMTs elicited an increased release of three of the four selected miRNAs (miR-199a-5p, miR-99b-5p and miR-125a-5p). This demonstrates that the panel of miRNAs is released in vitro during fibrosis by cell lines and primary cells. However, samples from patients are required to determine if these markers can also be implemented in a clinical setting to assess the dynamic range and specificity of the assay.

### 3.4. miR-199a-5p, miR-214-3p and miR-99b-5p Promote hTERT-HSC Activation

To establish a potential functional association between the selected miRNAs and fibrosis, we evaluated the effect of the four miRNAs on stellate cells, specifically in promoting HSC activation, proliferation, and migration.

TGF-β1 is known to upregulate miR-199a-5p and decrease CAV1 in HSCs. CAV1 in turn internalizes TGF receptors into caveolae and this internalization represses the TGF-β signaling pathway [76], thereby establishing an important link between miR-199a-5p and CAV1 in liver fibrosis progression [74]. Moreover, a recent publication by Yang et al., using primary rat HSCs and the immortalized human HSC cell line LX2, demonstrated that miR-199a-3p transfection resulted in inhibition of CAV2 and TGFβRI levels, subsequently enhancing the TGF-β signaling pathway and promoting HSC activation [51]. A similar mechanism involving miR-199a-5p and CAV1 has been proposed to exacerbate lung fibrosis [50]. Interestingly, TargetScan results demonstrate that miR-199a-3p (produced from the precursors 3′ arm) did not target CAV1 but CAV2. miR-199a-5p, on the other hand, showed a target sequence for CAV1 but not for CAV2 (supplementary excel file). This shows the important distinction between the two mature miRNA species and the different targets they may have. Using hTERT-HSCs we demonstrate that CAV1 expression was decreased by both miR-199a-5p transfection and TGF-β1 treatment. These results, and those identified by Yang et al. [51], demonstrate the relevance of both miR-199a-5p and miR-199a-3p in HSC activation and fibrosis progression. We also detected increased αSMA in agreement with the findings of Yang et al. [51] The effect of miR-199a-5p on migration and proliferation has only been assessed in tumors, where miR-199a-5p was shown to reduce cell proliferation and promote apoptosis [77]. Contrarily, we observed that miR-199a-5p promoted migration and cell proliferation in the hTERT-HSCs without impacting cell viability (Figure S2). The increase in migration and proliferation are functional parameters that further support the pro-fibrotic effects of miR-199a-5p on HSC.

It has been shown that miR-214 is increased intracellularly during liver fibrosis [46] and that miR-214 plays a role in HSC activation, promoting αSMA production and promoting proliferation in LX2 cells [53]. We corroborate that miR-214 promoted αSMA production in hTERT-HSC and we also demonstrate that miR-214-3p-transfected HSC showed increased proliferation and migration, supporting the hypothesis that miR-214-3p plays a role in liver injury through HSC activation, proliferation and migration. miR-99b-5p has been shown to play a role in wound healing and regulating cell proliferation and cell migration [58,59,78]. Interestingly, Turcatel et al. demonstrated using murine mammary gland cells that inhibition of miR-99b-5p decreased TGF-β activity by inhibiting SMAD3 phosphorylation [79], suggesting that miR-99b-5p is involved in the regulation of TGF-β signaling. Here, we identified that miR-99b-5p can elicit an increase in αSMA expression by HSC and also foster cellular migration and proliferation [79]. Finally, miR-125a-5p is released during chronic hepatitis B and serum concentrations of miR-125a-5p show a strong correlation to liver fibrosis staging [55]. miR-125a-5p has been shown to negatively regulate cell migration and proliferation as well as suppress tumor activity [80,81]. It has also been shown that TGF-β is capable of inducing an upregulation of miR-125a-5p in HBV-infected hepatocytes. Interestingly, downregulation of miR-125a-5p prevented activation of HSCs in vitro as suggested by Li et al. [56]. Following transfection of hTERT-HSC with miR-125a-5p, we observed no changes in αSMA expression, proliferation and migration.

## 4. Materials and Methods

### 4.1. Human Cell Lines

All cells were cultured at 37 °C in 5% CO_2_. HepaRG cells (Biopredic International, Saint-Grégoire, France) were seeded at 1 × 10^5^ undifferentiated cells/cm^2^ in basal medium with growth supplements ADD710 (Biopredic) and cultured for 14 days before differentiation. Cell differentiation was induced with basal medium containing differentiation supplements ADD720 (Biopredic) for 14 days and maintained for up to 4 weeks. HepaRG were passaged using Trypsin-EDTA (Invitrogen, Waltham, MA, USA, 25300). HepaRG were used at passages below 20.

hTERT-HSC were kindly provided by Dr. Bernd Schnabl (UC San Diego, San Diego, CA, USA) and were cultured in DMEM High Glucose (Invitrogen, 41965) supplemented with 10% fetal bovine serum (FBS) (Invitrogen, 10270) and 1% P/S (Gibco, Amarillo, TX, USA, 15070063). hTERT-HSC were passaged using Trypsin-EDTA. hTERT-HSC were maintained at low passages (<10) to avoid spontaneous activation.

THP-1 monocytic cells (Cell Line Service) were cultured at 2–10 × 10^5^ cells/mL in RPMI 1640 Medium (Bioconcept, Allschwil, Switzerland, 1-41F50-I) containing 10% FBS and 1% P/S. THP-1 cells were differentiated into macrophages over 48 h in RPMI 1640 Medium containing 100 ng/mL phorbol 12-myristate 12-acetate (PMA) (Sigma, St. Louis, MO, USA, 79346) as described in previous literature [50]. The differentiated THP-1 were washed with fresh medium and maintained in DMEM High Glucose supplemented with 10% FBS and 1% P/S. Differentiated THP-1 were detached using Accutase (Sigma, SCR005). THP-1 were used at passages below 30.

### 4.2. Generation of Microtissues

All co-culture MTs were generated using the MicroTissues 3D Petri Dish system from Sigma-Aldrich (Z764051-6EA) using UltraPure Agarose (ThermoFischer, Waltham, MA, USA, 16500100). Differentiated HepaRG, differentiated THP-1 and hTERT-HSC were counted and resuspended in William’s E Medium + GlutaMAX (Invitrogen, 32551), 5 μg/mL insulin, 5 μg/mL transferrin, 5 ng/mL sodium selenite (Sigma, 11074547001), 100nM dexamethasone (Sigma, D1756), 20% FBS and 1% P/S. After 72 h of aggregation the MTs were maintained/treated in an FBS-free version of the medium. Human liver MTs were generated using 2000 cells per MT, containing differentiated HepaRG, hTERT-HSC and differentiated THP-1.

Primary human microtissues (PHMTs) were generated using cryopreserved PHH (LOT: S1518T) and non-parenchymal cells (NPCs) (S1512T) (obtained from KalyCell, Plobsheim, France) from the same donor. Cells were thawed and seeded at a ratio of 2:1 (PHH:NPC) at 2000 cells per MT in the 3D Petri Dish system. MTs were allowed to aggregate in Williams E Medium + GlutaMax, 1% P/S, 10 μg/mL insulin, 5.5 μg/mL transferrin, 6.7 ng/mL sodium selenite (Sigma, I3146-5ML), 100 nM dexamethasone and 10% FBS. After spheroids were sufficiently compacted (day 10) the medium was exchanged to an FBS-free version of the medium, which was used for cell treatments. 

### 4.3. Induction of CYP3A4

For functional characterization, CYP3A4 induction was carried out using 20 µM rifampicin (RIF, Sigma, R3501) directly after the aggregation phase of the MTs medium. RIF treatment was refreshed at 24 h and ended at 48 h. Following this, the induced MTs were either tested for CYP3A4 activity and gene or protein expression as described below.

### 4.4. Cytochrome P450 Assay

P450-GloTM CYP3A4 Assay with Luciferin-IPA (Promega, Madison, WI, USA, V9001) was used to determine CYP3A4 activity. The P450-GloTM assay was carried out as described in the manufacturer’s protocols and adjusted to quantities necessary for the MTs. The luminescence was measured at 1000 m/s using a FlexStation 3 Microplate Reader (Molecular Devices, San Jose, CA, USA).

### 4.5. HPLC-MS/MS

Substrate conversion by MTs was determined in RIF-treated and untreated MTs by measuring the conversion of 25 µg/mL testosterone (Sigma, 86500) to hydroxytestosterone over time. Cell culture supernatant was collected at 6, 24 and 48 h and diluted in water supplemented with 2 mM ammoniumfluoride solution, and measurements were performed using HPLC-MS/MS (Agilent Technologies LC unit: Agilent Technologies, Santa Clara, CA, USA, 1100; MS detector: Agilent Technologies, 6410; columns SB-C8, Zorbax, 4.6 × 50 mm, 1.8 mm, Agilent Technologies). The column heater was set at 40 °C and the injection volume was set at 1 µL. Tandem MS was MS/MS (Agilent, Triple Quad, 6410), mode: ESI, MRM and positive source. Calibration standards were made of 1, 5, 10, 50 and 100 µg/mL testosterone in water supplemented with 2 mM ammonium fluoride. The absolute concentration of testosterone was calculated based on the calibration standards. Production of hydroxytestosterone was qualitatively assessed by determining the area under the curve.

### 4.6. Cell Treatments

Co-cultures (MTs) were formed as described above and treatment was carried out using maintenance medium. MTs were treated with methotrexate (MTX, Sigma, M8307), using the concentration range of 3.75–120 µM, and acetaminophen (APAP, Sigma, A5000), using the concentration range of 1–16 mM. Medium with or without substances was exchanged every 2–3 days. Subsequent experiments were performed with concentrations of 2 and 4 mM for APAP and 30 and 60 µM for MTX. In addition, 1 ng/mL TGF-β1 (Sigma, T5050), which is known to cause hepatocellular injury and to elicit a pro-fibrotic effect, was used as a positive control for fibrosis characterization.

### 4.7. Cell Viability Assay

Cell viability was assessed using the CellTiter-Glo^®^ Luminescent Cell Viability Assay (Promega, G7570, Madison, WI, USA). The assay was carried out as described in the manufacturer’s protocols and adjusted to quantities necessary for the MTs. 

### 4.8. Gene Expression Analysis

MTs were collected and washed using PBS then lysed using Qiazol Lysis Reagent (Qiagen, Hilden, Germany, 79306). mRNA was isolated following standard TRIzol extraction procedure with the addition of Glycogen (ThermoFisher, LT-02241, Waltham, MA, USA). RNA was reverse transcribed using M-MLV Reverse Transcriptase (Promega, M1705) and oligo dT (Qiagen, 79237, Hilden, Germany), and real-time PCR was performed using FastStart TaqMan^®^ Probe Master (Roche, Basel, Switzerland, 04673417001) and TaqMan probes from Invitrogen. Real-time TaqMan PCR was performed on selected genes (see Table S1). The qRT-PCR program used 10 min denaturation at 95 °C, followed by 40 cycles of 15 s at 95 °C and 1 min at 60 °C. The Ct values were generated using the Corbett Rotorgene Analysis Software 6000 and processed on GraphPad Prism. Beta-2-Microglobulin was used as an internal standard for the normalization. The Ct values were generated using the Corbett Rotorgene Analysis Software 6000 and processed on GraphPad Prism, and data are expressed as fold change.

### 4.9. Next-Generation Sequencing

MTs were formed as described above and treatment was carried out using maintenance medium. Cell culture supernatant was collected from untreated MTs and treated MTs for MTX 30 µM and APAP 2 mM and sent to GenXPro GmbH (Goethe-Universität Frankfurt am Main). miRNA was extracted from 1 mL of cell culture supernatant of two biological replicates and processed using the TrueQuant method which contains “Unique Molecular Identifiers” (UMIs) to avoid uneven amplification and artefact generation during the PCR stage. Data were initially processed by GenXPro to assign sequences to specific miRNAs and provide miRNA counts. Following this, data were processed in R using packages Bioconductor, DESeq2 and edgeR. Counts were initially normalized using a TMM method and differential miRNAs were calculated using edgeR with a GLM fit. PCA analysis was carried out using R and all data were plotted using ggplot2. miRNAs specific to MTX-induced fibrosis from our model were cross-checked with the publication by Krauskopf et al. [33] to confirm the specificity using clinical data from patients with APAP-induced toxicity. miRNAs were run through DIANA-miRpath v3.0 to identify potential links to fibrosis and targets to obtain the most promising miRNA panel to have fibrosis specificity.

### 4.10. miRNA Analysis

miRNeasy Serum/Plasma Kit (Qiagen,217184) was used for miRNA extraction of 180 μL medium reverse transcription and q-RT-PCR was carried out using TaqMan MicroRNA Reverse Transcription Kit (ThermoFischer, 4366596) and qRT-PCR Master Mix (ThermoFischer, 4444557). TaqMan q-RT-PCR primers were purchased from Invitrogen (Table S2). The reaction mix was prepared according to the manufacturer’s instructions for a final reaction volume of 10 μL with 3 μL miRNA extract. The PCR conditions were set for 30 min at 16 °C followed for 30 min at 42 °C and 5 min at 85 °C. All values were expressed as fold change.

### 4.11. Immunohistochemistry

3D Immunostaining: MTs were fixed with 4% PFA 1h and then washed in PBS + calcium and magnesium (PBS + Mg^2+^ and Ca^2+^). Fixed MTs stained fully using the protocol described by Ravenscroft et al. [78] with primary and secondary antibodies listed below (Table S3). Images were taken using an Olympus FV3000 confocal microscope.

2D Immunostaining: Cells were fixed in 4% PFA for 15 min and then washed with PBS + Mg^2+^ and Ca^2+^. Cells were permeabilized for 15 min using 0.1% Triton-X100 and then blocked in 1% BSA (Sigma, A2153) for 1 h at RT. Primary antibodies αSMA, ki67 and CAV1 (Table S3) were diluted in 1% BSA and incubated overnight at 4 °C. Cells were washed 3× with PBS and the secondary antibody diluted in 1% BSA was added for 1 h at RT. Cells were washed again and then counterstained with DAPI (Sigma, 10236276001) for 5 min, and then washed and imaged using the Zeiss Colibri 7 LED Fluorescence system or Olympus FV3000 confocal microscope.

### 4.12. Enzyme-Linked Immunosorbent Assay

Medium for protein determination was pooled over the whole treatment period. Human albumin ELISA (Bethyl Laboratories, Montgomery, TX, USA, E80-129). All buffers and solutions were made according to the buffer preparation guidelines. High-binding flat-bottomed plates (Greiner Bio-One, Kremsmünster, Austria, 655 061) were used for the ELISA. ELISA results were calculated using a four-parameter fit using the SoftMax Pro software.

### 4.13. Transfection of miRNA Mimics

hTERT-HSC were plated in 96-well plates (2000 cells/well), 48-well plates (10,000 cells/well) or 6-well plates (60,000 cells/well) and allowed to adhere overnight. HiPerfect Reagent was combined with 40 or 80 nM of the specific miRCURY LNA miRNA Mimic from Qiagen (labelled with 5’FAM) and mixed with DMEM High Glucose (no supplements), vortexed briefly and incubated at RT for 20 min. Following this the transfection mixture (composition in Table 2) was placed into each well containing ≈175 µL (96-well), ≈375 µL (48-well) or ≈1400 µL (6-well) of DMEM with 10% FBS and 1% P/S and the cells were incubated at 37 °C and 5% CO_2_ for 72 h.

### 4.14. Migration/Wound Healing Assay

hTERT-HSCs were seeded at 10,000 cells per well of a 48-well plate, allowed to adhere overnight and then transfected as described above. Following transfection, a scratch was made in each well and fresh medium was added with or without treatments and imaged every h for 48 h in the cellVivo incubation system by Olympus at 37 °C, 5% CO_2_ in a humidified chamber. Data were analyzed in ImageJ using the “Wound_healing_size_tool” (author: Volker Baecker), which allows batch analysis of the stack of 48 images for each condition. 

### 4.15. Western Blot

hTERT-HSC were seeded at 60,000 cells per well of a 6-well plate, allowed to adhere overnight and then transfected as described above. Following the 72 h transfection the cells were lysed in 100 µL of RIPA buffer with proteinase inhibitor (Thermofischer, 89900). Protein was quantified using a ROTI^®^Nanoquant Bradford assay (Bio-rad, Hercules, CA, USA, K880.1) using the 96-well format and a 1:40 dilution of sample. Lämmli SDS Gel with glycerin were prepared for the Bio-Rad Mini-Protean Vertical Electrophoresis Cell system using 0.75 mm gels. The gel was comprised of 12% resolving gel and 4% stacking gel, and was run using Lämmli running buffer for 45–60 min at 300 V, 2000 mA and 25 W. Samples were transferred onto a nitrocellulose membrane using Lämmli transfer buffer at 2 h at 20V, 100 mA, 20 W. The membrane was blocked overnight at 4 °C using the Intercept^®^ (TBS) Blocking Buffer (Li-Cor, Lincoln, NE, USA. 927-60001) diluted 1:1 in Tris-buffered saline (TBS). Primary and secondary antibodies were diluted in Intercept^®^ (TBS) Blocking Buffer diluted 1:1 in TBS + 0.2% Tween20 (TBS-T). Primary CAV1 (abcam, Cambridge, UK, ab2910) and β-actin (Santa Cruz, Sc-477778) antibodies were diluted 1:1000 and 1:200, respectively, and added to the membrane for 3 h at RT on a rocker and then washed 3× for 5 min using TBS-T. Secondary antibodies IRDye^®^ 680RD Donkey anti-Rabbit IgG Secondary Antibody (Li-Cor, 925-68073) and IRDye^®^ 800CW Goat anti-Mouse IgG Secondary Antibody (Li-Cor, 926-32210) were added at a dilution of 1:10,000 for 2 h at RT and the washing was repeated. Membrane was imaged on the Odyssey CLx Imaging system (Li-Cor) at 700 and 800 nm and intensity were measured using Image Studio Lite version 5.2 (Li-Cor).

### 4.16. Statistical Analysis

Data were analyzed using GraphPad Prism 8 (GraphPad Software, San Diego, CA, USA, Version 8.0.2) and expressed as mean values ± SD. The Student’s *t* test was used for comparison between two groups and one-way ANOVA was used for statistical analysis of multiple concentrations of the same treatment. *p* < 0.05 was considered to be significant: *, *p* ≤ 0.05; **, *p* ≤ 0.01; ***, *p* ≤ 0.001.

## 5. Conclusions

In conclusion, we demonstrate the ability of the MTs to respond to both chronic and acute DILI compounds, resulting in a phenotype that corresponds to clinical data. The MTs show basal and RIF-induced CYP3A4 activity and TGF-β1-treatment-induced fibrosis. We identified four miRNA biomarkers, which could be used to detect liver fibrosis, which we confirmed in both the cell line model and primary model, highlighting their suitability as in vitro models for assessing miRNA release during fibrosis.

Using miRNA mimics and hTERT-HSCs we were able to identify a link between three of the miRNAs (miR-199a-5p, miR-214-3p and miR-99b-5p) to HSC activation and two of the miRNAs (miR-199a-5p, miR-214-3p) to increased proliferation and migration. Finally, due to the specificity of the miRNAs and the link to HSC activation, we suggest these miRNAs, specifically miR-199a-5p, could be useful contributing biomarkers to a non-invasive panel for detecting fibrosis.

## Figures and Tables

**Table 1:** Literature used for miRNA selection.

miRNA	*In Vitro*	*In Vivo*	Clinical	Publications
miR-199a-5p	Increased in activated stellate cells. Mimic resulted in increased HSC activation. AntagomiR decreased HSC activation.	Links to lung fibrosis. Intracellular miR-199a levels correlate with liver fibrosis development. miR-199a is linked to ECM synthesis in CCl4 treated mice.	Increased in human fibrotic liver samples. Intracellular levels correlate with liver fibrosis development.	Cardenas et al., 2013 [50] Yang et al., 2020 [51] Ezhilarasan, 2018 [46] Roy et al., 2015 [52]
miR-214-3p	Intracellular miR-214 promotes HSC activation. Reduction in intracellular miR-214 ameloriates liver fibrosis.	Knockdown of intracellular miR-214 results in enhanced Sufu and decreased liver fibrosis. Upregulated intracellular miR-214 is overexpressed in mouse models resulting in liver fibrosis.	Intracellular miR-214 is linked to HCV induced liver fibrosis. Circulating miR-214 decreases in APAP overdose patients.	Ma et al., 2018 [53] Ezhilarasan, 2018 [46] Krauskopf et al., 2017 [32]
miR-125a-5p	Intracellular miR-125a is shown to be upregulated in activated HSCs.	miR-125a expression was increased in mouse livers with CCL4 induced fibrosis.	Intracellular miR-125a linked to liver fibrosis from HBV infection. Circulating miR-125a is linked to HCC.	Coppola et al., 2018 [54] Oura et al., 2019 [55] Li et al., 2016 [56]
miR-99b-5p	miR-99b expression is linked to dermal wound healing response using *in vitro* models. Involved in regulating cell proliferation and cell migration.	miR-99b expression in mice is linked to dermal wound healing response. miR-99b expression linked to spinal cord regeneration.	Expression of miR-99b is significantly associated with pericellular fibrosis from NAFLD.	Estep et al., 2010 [57] Jin et al., 2013 [58] Cao et al., 2017 [59]

**Table 2:** Reagent quantities for miRNA mimic transfection.

Plate Size	Cell Number	Concentration (mM)	HiPerfect Reagent	Mimic	DMEM without Supplements	Final Medium + Reagent Quantity per Well
96-well	2000	40	0.75 µL	0.12 µL	24.25 µL	200 µL
		80	0.75 µL	0.24 µL	24.25 µL	
48-well	10,000	40	1.5 µL	0.24 µL	48.5 µL	400 µL
		80	1.5 µL	0.48 µL	48.5 µL	
6-well	60,000	40	6 µL	0.96 µL	194 µL	1600 µL
		80	6 µL	1.92 µL	194 µL	

## Data Availability

miRNA sequencing data can be found at https://doi.org/10.5281/zenodo.5482199.

## Supplementary Materials

The following are available online at https://www.mdpi.com/article/10.3390/ijms22189799/s1.

## Abbreviations

ALP	Alkaline phosphatase
ALT	Alanine aminotransferase
AOP	Adverse outcome pathway
APAP	Acetaminophen
AST	Aspartate aminotransferase
CAV1	Caveolin-1
CAV2	Caveolin-2
Col 4	Collagen IV alpha 1
Col I	Collagen I alpha 1
CYP	Cytochrome P450
ECM	Extracellular matrix
FBS	Fetal bovine serum
FN1	Fibronectin
GSH	Glutathione
HSC	Hepatic stellate cell
hTERT-HSC	Immortal activated human hepatic stellate cells
miRNA	Micro RNA
mRNA	Messenger RNA
MTs	Microtissues
MTX	Methotrexate
NPC	Non-parenchymal cells
PFA	Paraformaldehyde
PHH	Primary human hepatocytes
PHMTs	Primary human microtissues
PMA	Phorbol 12-myristate 12-acetate
RIF	Rifampicin
TAA	Thioacetamide
TBL	Total bilirubin
TGF-β1	Transforming growth factor-β
TNF-α	Tumour necrosis factor alpha
UMI	Unique Molecular Identifiers
αSMA	α-smooth muscle actin
