# Supplementary material for: Identification of miR-199a-5p, miR-214-3p and miR-99b-5p as Fibrosis-Specific Extracellular Biomarkers and Promoters of HSC Activation
Source: Int J Mol Sci. 2021 Sep 10;22(18):9799. doi: 10.3390/ijms22189799 (PMC8464755; doi:10.3390/ijms22189799)
Supplement: Supplementary file 1 [file ijms-22-09799-s001.zip › ijms-1338035-SM - for publish.pdf]

International Journal of *Molecular Sciences*

Supplementary Materials

Identification of miR-199a-5p, miR-214-3p and miR-99b-5p as Fibrosis-Specific Extracellular Biomarkers and Promoters of HSC Activation

Catherine Jane Messner [1,2,3,*], Saskia Schmidt [1,2], Dilek Özkul [1], Carine Gaiser [1], Luigi Terracciano [4], Stephan Krähenbühl [2] and Laura Suter-Dick [1,3,*]

1 School of Life Sciences, University of Applied Sciences and Arts Northwestern Switzerland, Muttenz, Hofackerstrasse 30, CH-4132, Switzerland; saskia.schmidt@fhnw.ch (S.S.); dilek.oezkul@gmail.com (D.Ö.); carine.gaiser@fhnw.ch (C.G.)
2 Department of Pharmaceutical Sciences, University of Basel, Basel CH-4001, Switzerland; Stephan.Kraehenbuehl@usb.ch
3 Swiss Centre for Applied Human Toxicology (SCAHT), Basel, Switzerland
4 Institute of Pathology, University Hospital Basel, Basel, Switzerland; Luigi.Terracciano@usb.ch
* Correspondence: catherine.messner@fhnw.ch (C.J.M.); laura.suterdick@fhnw.ch (L.S.-D.)

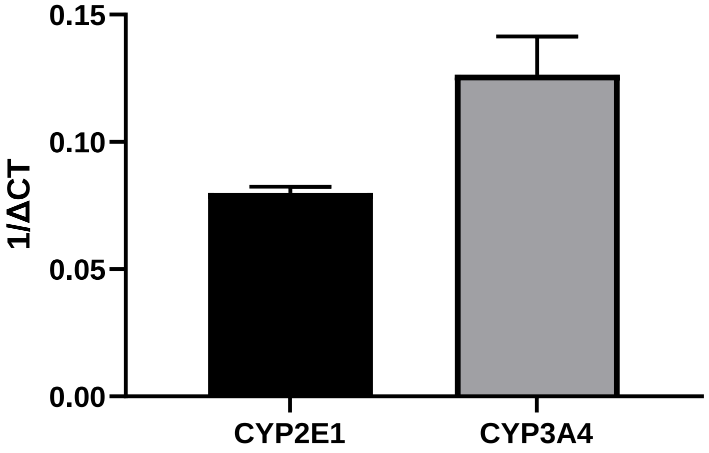

Figure S1. Basal CYP3A4 and CYP2E1 expression in multicellular MTs**.** MTs were generated and allowed to aggregate over 72 h. MTs were cultured in maintenance medium and collected at 72 h and 14 days to investigate the gene expression of CYP3A4 (data shown as fold change in figure 1) and CYP2E1, respectively. Gene expression was measured using q-RT-PCR as described in material and methods, probes are listed in table S1. Data are expressed as relative expression calculated as 1/ΔCT, normalized to B2M (n = 3).

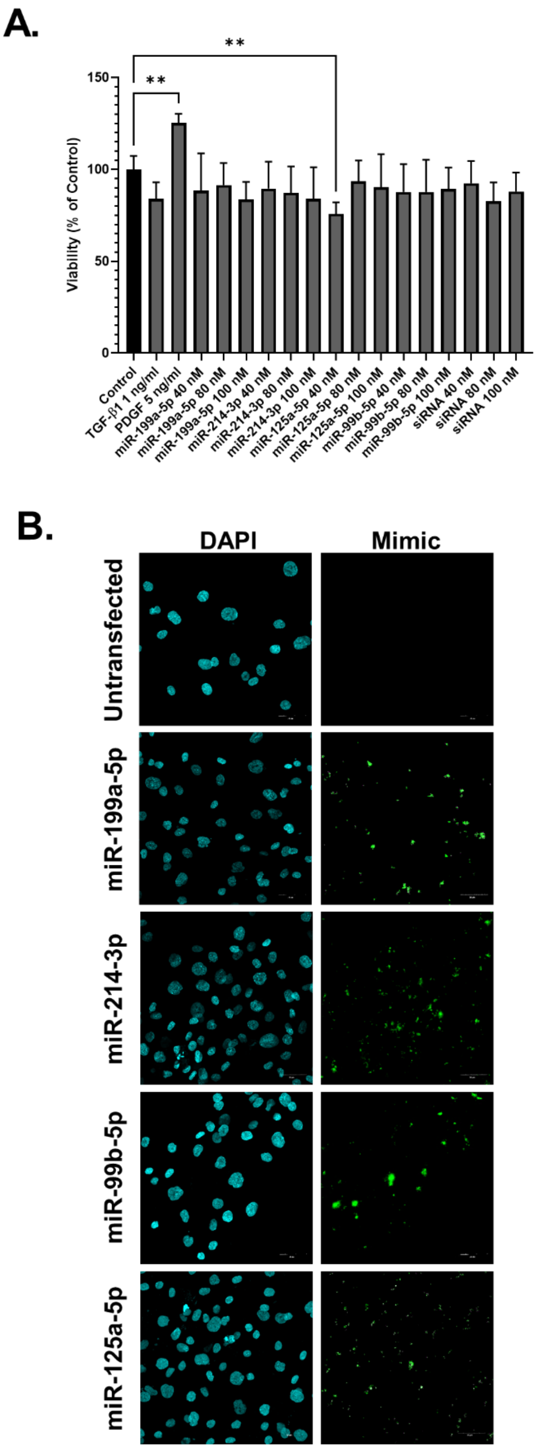

Figure S2. hTERT-HSCs transfection viability and confirmation. hTERT-HSCs were either treated (TGF-β1 or PDGF) or transfected with different concentrations of the four miRNAs of interest miR-199a-5p, miR-214-3p, miR-125a-5p and miR-99b-5p. Following 72 h of treatment/transfection the viability was measured using the cell-counting kit 8 (Sigma, 96992) compared to the untreated control. $n = 6$. One-way ANOVA with multiple comparisons; **, $p \leq 0.01$ (**A**). Mimics were labelled with 5-FAM so we could confirm successful transfection microscopically. Images were taken at the Olympus confocal following fixation with 4% PFA and washing with PBS (**B**).

Table S1. TaqMan primers used for gene expression analysis by q-RT-PCR.

Gene of Interest	Abbreviation	Invitrogen Ref. Nr
Beta-2-Microglobulin	B2M	Hs00187842_m1
Actin, alpha 2, smooth muscle	ACTA2 (αSMA)	Hs00426835_g1
Collagen 1 alpha 1	COL1α1	Hs00164004_m1
Collagen 4 alpha 1	COL4α1	Hs00266237_m1
Cytochrome P450 3A4	CYP3A4	Hs04260376_m1
Cytochrome P450 2E1	CYP2E1	Hs00559368_m1

Table S2. TaqMan primers used for miRNA analysis by q-RT-PCR.

miRNA of Interest	Assay Name	Assay Number
Cel-miR-39/Spike-in	Cel-miR-39-3p	000200
RNU44	RNU44	001094
miR-122-5p	hsa-miR-122-5p	002245
miR-199a-5p	hsa-miR-199a-5p	000498
miR-214-3p	hsa-miR-214-3p	002306
miR-125a-5p	hsa-miR-125a-5p	002198
miR-99b-5p	hsa-miR-99b-5p	000436

Table S3. Antibodies used for immunohistochemistry.

Protein of Interest	Primary Antibody	Secondary Antibody
αSMA (ACTA2)	Mouse polyclonal antibody (Sigma A5228)/1:200	Goat anti mouse Alexafluor 488 (A-11017)/1:1000
Collagen I	Mouse monoclonal antibody (Abcam ab90395)/1:100	Goat anti mouse Alexafluor 488 (A-11017)/1:1000
Fibronectin	Rabbit polyclonal antibody (Abcam ab32457)/1:100	Goat anti rabbit Alexafluor 546 (A-11071)/1:1000
Albumin	Rabbit monoclonal antibody (Abcam ab207327)/1:800	Goat anti rabbit Alexafluor 546 (A-11071)/1:1000
Vimentin	Rabbit monoclonal antibody (Abcam ab92547)/1:100	Goat anti rabbit Alexafluor 546 (A-11071)/ 1:1000
CYP3A4	Rabbit polyclonal antibody (Sigma, AB1254)/1:100	Goat anti rabbit Alexafluor 488 (A-11070)/1:1000
CD68	Mouse monoclonal antibody (Abcam ab955)/1:100	Goat anti mouse Alexafluor 488 (A-11017)/1:1000
CAV1	Rabbit polyclonal (abcam, ab2910)/1:500	Goat anti rabbit Alexafluor 546 (A-11071)/1:1000
Ki67	Rabbit polyclonal antibody (abcam, ab15580)/1:300	Goat anti rabbit Alexafluor 546 (A-11071)/1:1000
